# A Systems Pharmacology Approach for Identifying the Multiple Mechanisms of Action for the Rougui-Fuzi Herb Pair in the Treatment of Cardiocerebral Vascular Diseases

**DOI:** 10.1155/2020/5196302

**Published:** 2020-01-08

**Authors:** Chun Li, Xia Du, Yang Liu, Qi-Qi Liu, Wen Bing Zhi, Chun Liu Wang, Jie Zhou, Ye Li, Hong Zhang

**Affiliations:** ^1^Pharmacy College, Shaanxi University of Chinese Medicine, Xianyang, Shaanxi, China; ^2^Shaanxi Academy of Traditional Chinese Medicine, Xi'an, Shaanxi, China

## Abstract

Cardiocerebral vascular diseases (CCVDs) are the main reasons for high morbidity and mortality all over the world, including atherosclerosis, hypertension, myocardial infarction, stroke, and so on. Chinese herbs pair of the *Cinnamomum cassia Presl* (Chinese name, rougui) and the *Aconitum carmichaelii Debx* (Chinese name, fuzi) can be effective in CCVDs, which is recorded in the ancient classic book *Shennong Bencao Jing*, *Mingyibielu* and *Thousand Golden Prescriptions*. However, the active ingredients and the molecular mechanisms of rougui-fuzi in treatment of CCVDs are still unclear. This study was designed to apply a system pharmacology approach to reveal the molecular mechanisms of the rougui-fuzi anti-CCVDs. The 163 candidate compounds were retrieved from Traditional Chinese Medicine System Pharmacology Database and Analysis Platform (TCMSP). And 84 potential active compounds and the corresponding 42 targets were obtained from systematic model. The underlying mechanisms of the therapeutic effect for rougui-fuzi were investigated with gene ontology (GO) enrichment analysis and Kyoto Encyclopedia of Genes and Genomes (KEGG) pathway enrichment analysis. Then, component-target-disease (C-T-D) and target-pathway (T-P) networks were constructed to further dissect the core pathways, potential targets, and active compounds in treatment of CCVDs for rougui-fuzi. We also constituted protein-protein in interaction (PPI) network by the reflect target protein of the crucial pathways against CCVDs. As a result, 21 key compounds, 8 key targets, and 3 key pathways were obtained for rougui-fuzi. Afterwards, molecular docking was performed to validate the reliability of the interactions between some compounds and their corresponding targets. Finally, UPLC-Q-Exactive-MS^E^ and GC-MS/MS were analyzed to detect the active ingredients of rougui-fuzi. Our results may provide a new approach to clarify the molecular mechanisms of Chinese herb pair in treatment with CCVDs at a systematic level.

## 1. Introduction

Cardiocerebral vascular diseases (CCVDs), including thrombosis, atherosclerosis, hypertension, cardiac hypertrophy, myocardial infarction, heart failure, stroke, and cerebral ischemia, are the major health problem all over the world, claiming more than 17.3 million lives annually [1–3]. Given the high incidence and high mortality of CCVDs, seeking feasible prevention and treatment strategy is highly imperative and critical to human health [4, 5]. Western medicine is currently the mainstream medical treatment for CCVDs. However, some certainly existing ill-effects are still unavoidable. For example, some antihypertensive drugs have triggered serious side effects, with hot flush, fatigue, shortness of breath, headache, dizziness rhabdomyolysis, and hepatic diseases [6–8]. Therefore, it is very indispensable to seek better, safe, and effective treatment strategies in treatment of CCVDs.

Herb pairs, the simplest form and the centralized representative of Chinese herbal compatibility, intrinsically convey the basic idea of traditional Chinese medicines' prescriptions that has attracted comprehensive attention for their ability to treat complex and chronic diseases due to their moderate treatment effect and lower side effect [9–11]. The herb pair (rougui-fuzi) consists of the twigs of *Cinnamomum cassia Presl* (Chinese name, rougui) and the root of *Aconitum carmichaelii Debx* (Chinese name, fuzi) that can be effective in the treatment of CCVDs, and usually in a proportion of 1 : 1, according to the classic ancient book *Shennong Bencao Jing*, *Mingyibielu* and *Thousand Golden prescriptions* [12]. Among them, the herb *Cinnamomum cassia Presl* can increase coronary blood flow, improve coronary circulation and myocardial nutritional status, and was often used in the prevention and treatment of coronary heart, arrhythmia, rheumatic heart disease, cardiogenic hypertension, and cardiovascular diseases [13–16]. *Aconitum carmichaelii Debx* has the functions of protecting cardiac muscle cell, fight arrhythmia, decrease inflammatory reaction, and inhibit tumor. Moreover, several active constituents of these two herbs are documented to exhibit various biological activities, which contribute to the CCVDs. For example, cinnamaldehyde, an index component of the *Cinnamomum cassia Presl*, can dilate peripheral blood vessels, improve blood circulation at the end of blood vessels, and improve myocardial blood supply. The aconite polysaccharide could increase the synthesis of metallothionein, reduce the production of malondialdehyde (MDA) and the release of lactate dehydrogenase (LDH), and inhibit cardiomyopathy apoptosis [17]. As is demonstrated above, the herb pair rougui-fuzi plays an important role in the treatment of CCVDs, but molecular mechanisms of rougui-fuzi in treatment of CCVDs are still indecisive.

Systems pharmacology has become a powerful tool to systematically reveal the function and behavior of a complex biological system, and it has become a novel and efficient way to explore the pharmacological mechanism of traditional Chinese medicine [18, 19]. It likewise has been commonly presented lately for exploring the molecular mechanisms of traditional Chinese medicine and different complex chronic health conditions, including CCVDs, cancer, and metabolic illness [20, 21]. It is more remarkable that systems pharmacology was also used to reveal the therapeutic mechanism of herb pairs in treatment with disease. For example, the action of herb pair (danggui-honghua) in treatment of blood stasis syndrome (BBS) through interactions between multigenes and multicompounds, instead of directly acting on either single target or single constituent, was to successfully elucidate the polypharmacological mechanisms underlying the efficiency of danggui-honghua for BSS treatment based on systems pharmacology [22]. Considering the herbal constituents and their related targets based on systems pharmacology analysis can enable us to better comprehend the molecular mechanisms of the herb pair rougui-fuzi [23].

**Figure 1:**
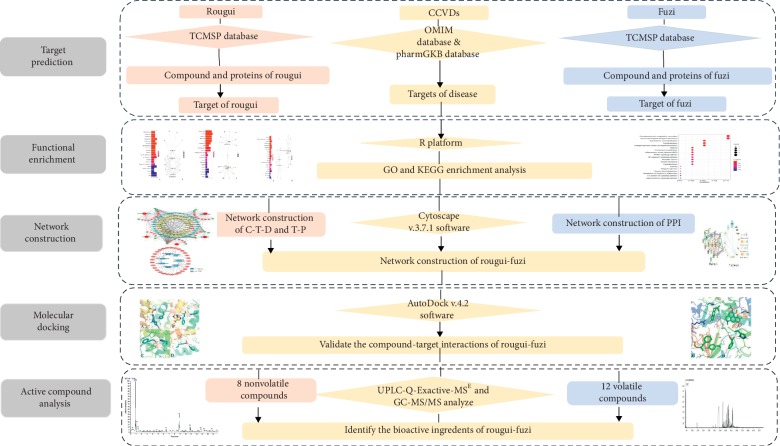
Flowchart of the experimental procedures.

In this study, information on constituents and corresponding candidate targets of rougui-fuzi was acquired from TCMSP databases. Then, the related biological processes and signal pathways were obtained according to GO and KEGG enrichment analysis. Moreover, compound-target-disease (C-T-D) and target-pathway (T-P) networks were established. Meanwhile, protein-protein interaction (PPI) was mapped using important pathway-related targets for screening core target proteins based on (STRING) database. After that, molecular docking was carried out to verify the reliability of compound-target binding interactions. Finally, to further determine rougui-fuzi components, the UPLC-Q-Exactive-MS^E^ and GC-MS/MS analyses were performed (the whole flowchart has been shown in Figure 1). Overall, this work may provide a useful system pharmacology framework to interpret multicomponent, multitarget, multichannel mechanism for the herb pair rougui-fuzi, and may give some basis and enlightenment in the further research for the herb pair.

## 2. Materials and Methods

### 2.1. Compound Database Construction for Rougui-Fuzi

The constituents of medicinal herbs *Aconitum carmichaelii Debx* and *Cinnamomum cassia Presl* were extracted from the Traditional Chinese Medicine System Pharmacology Database and Analysis Platform (TCMSP, http://lsp.nwu.edu.cn/tcmsp.php) [24, 25]. TCMSP is a unique system pharmacology platform designed for herbal medicines, which provides up-to-date, quantitative, and accurate structural and physicochemical properties such as drug targets and their relationships with diseases. Meanwhile, a large-scale structural information (29,384 chemicals in total with 13,144 unique molecules) with manually curated information for all recorded herbs in Chinese pharmacopoeia was integrated [26].

### 2.2. Target Fishing

Drug target indentifying is momentous to elucidating the biological basis of traditional Chinese medicine. Thus, in this work, we applied a systematic model that efficiently enriched chemical, genomic, and pharmacological information for drug target, based on random forest (RF) and support vector machine (SVM) methods. The robust model showed optimal performance of predicting the drug-target interactions, with 82.83% concordance, 81.33% sensitivity, and 93.62% specificity, which calculated the possibility of interactions between each ingredient and its target from TCMSP [27, 28]. There is a nonstandard problem in the naming of compound targets searched in the database; so, all TCMSP drug targets are imported into the UniProt (https://www.uniprot.org/) database, the target gene name is entered to define the species as “*Homo sapiens*,” and all protein names are corrected to their official name (official symbol). The human CCVD-related diseases associated with genes were secured from the curative targets database such as the Online Mendelian Inheritance in Man (OMIM, http://www.omim.org/) and Pharmacogenomics Knowledgebase (PharmGKB; http://www.pharmgkb.org) [29–32]. The keywords were “stroke,” “anti-platelet aggregation,” “atherosclerosis,” “improving microcirculation,” and “antithrombin” [33–35].

Based on the above, the previous compound targets are mapped with the genes of human CCVD-correlative disease to obtain their common targets, for better deciphering the role of rougui-fuzi against CCVDs.

### 2.3. Functional Enrichment Analysis

To better comprehend the potential biological processes and pathways of rougui-fuzi in treatment of CCVDs, the Kyoto Encyclopedia of Genes and Genomes (KEGG) pathway (http://www.genome.jp/kegg/pathway.html) and gene ontology (GO) enrichment analyses were performed based on the ClusterProfiler software package on *R* platform, and compared with Visualization [36]. Afterwards, the GO interactive network and the bubble diagram of KEGG pathways were structured based on the topGO packet of *R* platform [37]. *P* value was calculated in these two enrichment analyses, and *P* < 0.05 suggested the enrichment degree of statistically significant [38]. As a result, the pathway association and GO functions based on their enrichment were found and appropriately described.

### 2.4. Network and Protein-Protein Interaction Data Construction

For better dissecting the potential molecular mechanism of rougui-fuzi, we established three corresponding networks: (1) compound-target-disease (C-T-D) network. Active constituents of rougui-fuzi, corresponding targets, and CCVD-related diseases were employed to generate the C-T-D network in which an ingredient and a target are connected with each other if this protein is a known or validated either target or disease of this molecule. (2) Target-pathway (T-P) network. We extracted the precise pathway information of targets from the database of KEGG, and then constructed a target-pathway bipartite graph that comprises targets and their corresponding normative pathways. The previous two were generated in Cytoscape 3.7.2 software that is a standard tool for biological network visualization and data integration to further analyze the mutual relations in the network [37, 39]. (3) Protein-protein interaction (PPI network) was mapped using important pathway-related targets for screening core target proteins based on (STRING) database (https://string-db.org/). The version 11.0 of STRING was employed to seek for the PPI data, with the species limited to “*Homo sapiens*” and a confidence score >0.4 [40]. Meanwhile, the node 1, node 2, and combined score of the STRING database were imported into Cytoscape3.7.1 software, with setting the node size and color to map the degree value and score value to construct PPI network [41].

### 2.5. Molecular Docking

To verify the reliability of compound-target binding interactions, molecular docking program was in progress using AutoDock software (version 4.2), which employs the Lamarckian genetic algorithm (LGA) for each progress [42]. All the 3D structures of target proteins were downloaded from the RCSB PDB database (http://www.pdb.org), so that proteins and ligands could be prepared in the AutoDock tools before performing the docking progress [43]. The auxiliary program Autogrid was used to generate the docking area, which was defined as a 60 × 60 × 60 3D grid centered around the ligand-binding site with a 0.375 Å grid space. All bond rotations for the ligands were ignored in this study. Finally, the docking binding energy was calculated to explore binding interactions between the compounds and their targets in our studies, the potential protein targets of TCM were successfully predicted with a good compound-protein binding affinity based on the threshold level of −5.0 kcal/mol [43].

### 2.6. UPLC-Q-Exactive-MSE Analysis of Rougui-Fuzi

To verify the potential ingredients of rougui-fuzi, the UPLC-Q-Exactive-MS^E^ analysis was applied. Some reagents were also used: rougui (batch no. 19061412) was provided by Shaanxi Kangyu Pharmaceutical Co., Ltd (Xi'an, China), and fuzi (batch no. 150816) was provided by Shaanxi Kangyu Pharmaceutical Co., Ltd (Xi'an, China). LC-MS-grade acetonitrile was obtained from Thermo Fisher Scientific (China) co., ltd. (Shanghai, China), MS-grade formic acid was obtained from Thermo Fisher Scientific (China) co., ltd. (Shanghai, China). The rougui-fuzi extracts were analyzed on a Thermo Fisher Scientific quadrupole-electrostatic field orbital well high-resolution system coupled with H-Class UPLC system. Separations were accomplished on an Accucore AQ C18 column (150 mm × 2.1 mm, 2.6 *μ*m). The mobile phase included 0.1% formic acid aqueous solution (Solvent A) and acetonitrile (Solvent B) and a gradient elution program was performed: 0 min, 98% solvent A; 17 min, 50% solvent A; 20 min, 98% solvent A. The flow rate was set at 0.3 mL/min. Column temperature was kept at 30°C and the total run time was 20 min. The autosampler was conditioned at 4°C and the injection volume was 1 *μ*L. The ion source of thermoelectric spray (HESI) mass spectrometry was used to collect data in full switch ion mode. The sheath gas flow rate was set 30 L/min, spray voltage was set 3.50 kV, and capillary temperature and aux gas heater temperature were 300°C. S-lens RF level was set 55 V. The scan mode was Full MS and dd-MS^2^, Full MS resolution of the detection mode was 7000, and the dd-MS^2^ resolution was 175000. The scan range was from 100 to 1200 Da. Leucine-enkephalin was used as the lock mass for generating reference ions in the positive and negative modes.

### 2.7. GC-MS/MS Analysis of Rougui-Fuzi

The rougui-fuzi extracts were analyzed on Shimadzu TQ8040 coupled with Shimadzu 2010Plus GC system. Separation was performed on a 19091s-433HP-5MS (30 m × 0.25 mm ID with 0.25 *μ*m) capillary column. The inlet temperature was set at 260°C; the carrier gas (helium; purity > 99.996%) was maintained at a constant flow of 1.0 mL·min^−1^; the mode of inlet was split less; the injection volume was 1 *μ*L; the temperature of column oven was programmed from an initial value of 60°C (hold for 10 min), and then raised at 4°C·min^−1^ up to 220°C (hold for 20 min); the split ratio was set 20 : 1.

## 3. Result and Discussion

### 3.1. Ingredient Collection of Rougui-Fuzi

In this research, a total of 165 compounds were candidate compounds extracted in rougui-fuzi, including 65 kinds in fuzi and 100 kinds in rougui. Among the 165 compounds, 2 were duplicated and therefore removed, resulting in a list of 163 components. Through observation and analysis, it was found that fuzi mainly includes alkaloids, flavonoids, polysaccharides, sterol, and organic acid [44]. Rougui mainly includes volatile oils, polyphenols, flavonoids, polysaccharides, lignin class, and diterpenes [14].

### 3.2. Target Prediction and Disease Mapping

In this study, a total of 194 genes were linked to the 163 identified compounds of the herb pair by TCMSP database. Among them, 65 ingredients in fuzi act together on 58 targets, 100 compounds in rougui act together on 181 targets, and there are 45 common targets in rougui-fuzi. It is potently demonstrated that the same active ingredients can act on different targets, and different active constituents can also act on the same target, reflecting its multicomponent, multitarget anti-CCVD mode of action. Then, through the OMIM and pharmGKB databases, 158 targets obtained were mapped to stroke-related drug targets, 547 targets obtained were reflected to antiplatelet aggregation-related drug targets, 232 targets obtained were mirrored to antiatherosclerosis-related drug targets, 479 targets obtained were reflected to improve microcirculation-related drug targets, and 79 targets obtained were mirrored to antithrombin-related drug targets. Finally, 84 potential active compounds (see [Supplementary-material supplementary-material-1]) and corresponding 42 targets (see [Supplementary-material supplementary-material-1]) were identified after deduplication for rougui-fuzi in treatment with CCVDs.

### 3.3. GO Term and KEGG Pathway of Potential Target Gene Enrichment Analysis

#### 3.3.1. GO Enrichment Analysis

**Figure 2:**
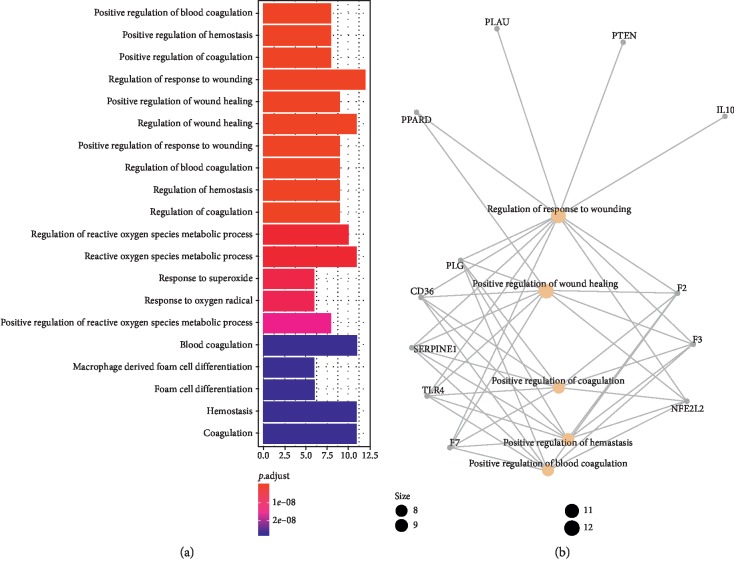
Biological process (BP) analysis of the predicted targets in rougui-fuzi. (a) The 30 GO terms for BP ontology with the most significant *P* values of targets in CCVDs. (b) The network diagram of core target regulation pathway.

**Figure 3:**
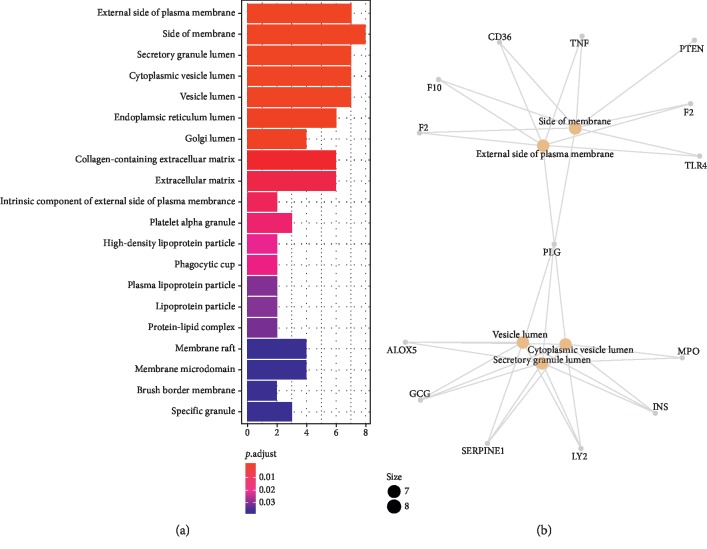
Cellular component (CC) analysis of the predicted targets in rougui-fuzi. (a) The 30 GO terms for CC ontology with the most significant *P* values of targets in CCVDs. (b) The network diagram of core target regulation pathway.

**Figure 4:**
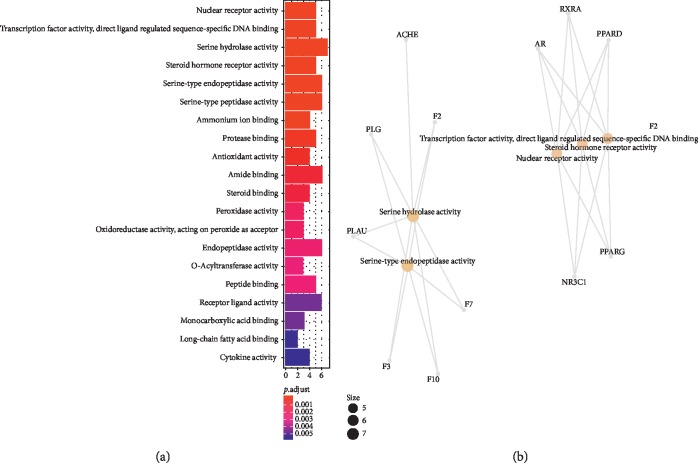
Molecular function (MF) analysis of the predicted targets in rougui-fuzi. (a) The 25 GO terms for MF ontology with the most significant *P* values of targets in CCVDs. (b) The network diagram of core target regulation pathway.

The results of GO evaluation were illustrated by the biological process (BP), cell component (CC), and molecular function (MF) terms. In the *R* package of clusterProfiler, 30 terms of BPs, 30 terms of CCs, and 25 terms of MFs enriched for these potential targets were recognized as *P* < 0.05. Depending on the outcomes of GO enrichment, the enriched BP ontologies were dominated by positive regulation of blood coagulation, positive regulation of hemostasis, positive regulation of wound healing, and regulation of response to wounding, indicating that the active components of rougui-fuzi interact primarily with related targets in the positive regulation (see Figure 2). The enriched CC ontologies were dominated by secretory granule, cytoplasmic vesicle lumen, vesicle lumen, external side of plasma membrane, and side of membrane (see Figure 3). The enriched MF ontologies were dominated by nuclear receptor activity, serine hydrolase activity, receptor ligand activity, transcription factor activity, direct ligand-regulated sequence-specific DNA binding, and steroid hormone receptor (see Figure 4).

### 3.4. KEGG Pathway Annotation Analysis

**Figure 5:**
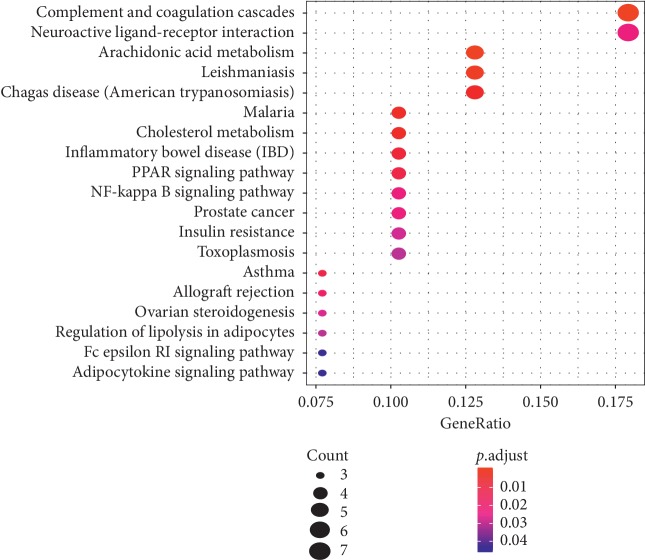
KEGG pathway annotation analysis of the rougui-fuzi predicted targets. Bubble diagram of the top 19 KEGG pathways.

The results showed that 42 targets were mapped to a total of 19 pathways (*P* < 0.05) through the bubble diagram (see Figure 5). Next, we excluded the CCVD-independent pathways such as “leishmaniasis,” “malaria,” and “inflammatory bowel disease,” and selected the most potential target enrichment of the pathways for analysis. It was not difficult to find that complement and coagulation cascades, neuroactive ligand-receptor interaction, and arachidonic acid metabolism are relatively significant pathways. Even though PI3K-Akt signaling pathway is not present in Figure 5, it plays an important role in the treatment of cardiovascular disease, and, the 6 differentially expressed genes involved in PI3K-Akt signaling pathway are as follows: IFNB1, IL4, INS, PTEN, RXRA, and TLR4, which have a crucial role in CCVD-related diseases [45, 46]. As a result, 7 pathways were determined in treatment of CCVDs.

### 3.5. Network Construction and Analysis

Centrality is a universal used notion in network analysis to express the significance of a node throughout the network. This work mainly measures the degree centrality (DC), betweenness centrality (BC), and closeness centrality (CC). The DC is the sum of the number of direct connections between a node and other nodes. It is the most direct metric to characterize the importance of nodes in network analysis. CC is the sum of the distances of a node to all other nodes, reflecting the extent of proximity of a node to other nodes. Betweenness centrality is the number of shortest paths passing through a node, reflecting the extent of cohesion of the nodes in the network. The greater the number of the shortest paths passing through a node, the higher its value [47].

### 3.6. Compound-Target-Disease Network and Analysis

**Figure 6:**
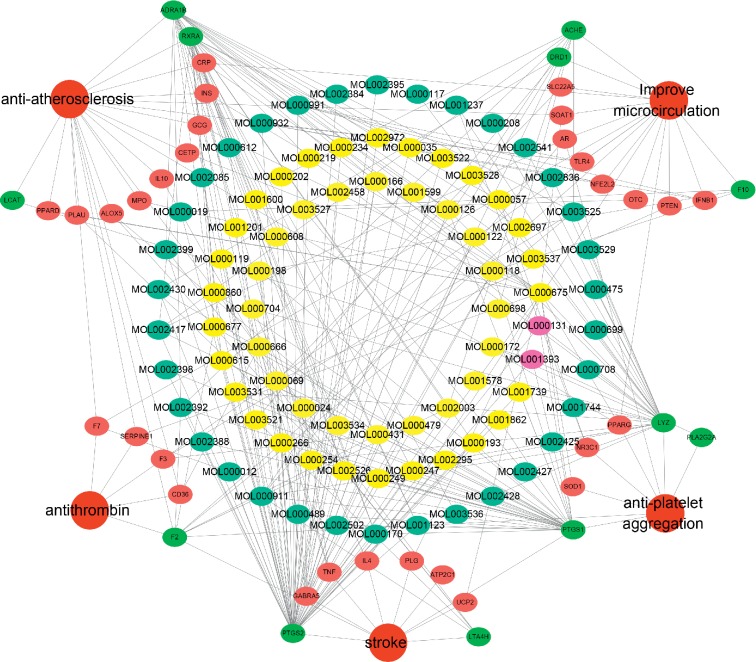
Compound-target-disease (C-T-D) network in treatment of CCVDs for rougui-fuzi. The yellow circle represented the ingredients of rougui, the blue circle represented the constituents of fuzi, and the purple circle represented the common compounds of the rougui-fuzi. The pink circle represents potential targets of all compounds in rougui and fuzi, the green circle the common targets the compound of the rougui-fuzi, and the red circle denoted the CCVD-related diseases.

In this work, C-T-D network was constructed including 131 nodes (84 active compounds, 42 potential targets, and 5 CCVD-related diseases) and 293 edges (the interactions between different nodes) (see Figure 6). The yellow circle represented the ingredients of rougui, the blue circle represented the constituents of fuzi, and the purple circle represented the common compounds of the rougui-fuzi. The pink circles represented potential targets of all compound in rougui and fuzi, the green circle the common targets the compound of the rougui-fuzi, and the red circle denoted the CCVD-related diseases. The results indicated that the active compounds had an average DC value of 2.7619, an average CC of 0.3464, and an average BC of 0.0068. Among them, the three centralities of 21 compounds were all higher than the average, indicating that these compounds have a relatively important status in the network (see Table 1). Here, oleic acid is one of the core components of rougui-fuzi and displays higher centralities of target interactions (DC = 17, CC = 0.4577, BC = 0.1394). It has been reported as a potential preventive and therapeutic CCVD safety reagent based on some experiments [48, 49]. In addition, palmitic acid [50] (DC = 6, CC = 0.0332, BC = 0.3779), eugenol [51] (DC = 5, CC = 0.0297, BC = 0.4305), coumarin [52, 53] (DC = 5, CC = 0.0127, BC = 0.3757), and linoleic acid [54] (DC = 4, CC = 0.0181, BC = 0.4140) also play an important role in anti-CCVDs.

### 3.7. Target-Pathway Network and Analysis

**Figure 7:**
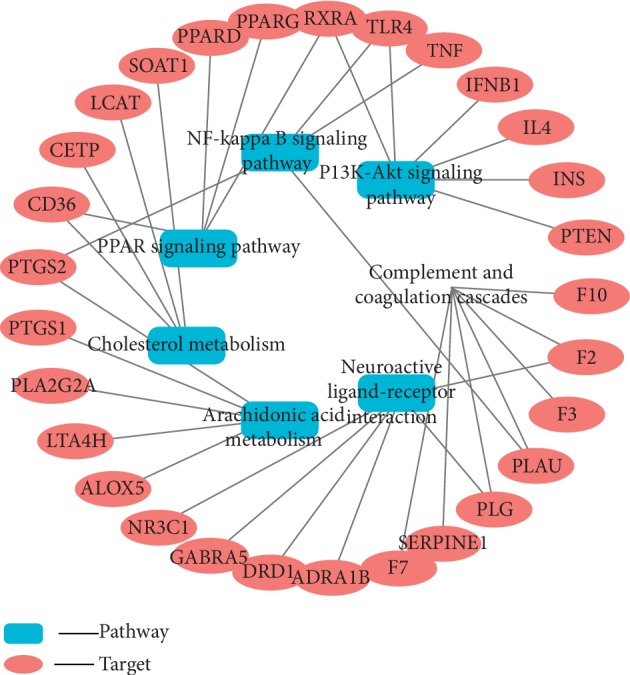
Target-pathways (T-P) network in treatment with CCVDs for rougui-fuzi. The pink ellipse represented the targets, and the blue rectangle represented the significant pathways of rougui-fuzi in treatment with CCVDs.

In this study, the target-pathway (T-P) network was constructed including 36 nodes (29 potential targets and 7 CCVD-related pathways) and 41 edges (see Figure 7). The pink ellipse represented the targets, and the blue rectangle represented the significant pathways of rougui-fuzi in treatment with CCVDs. The results indicated that the inherent pathways had an average DC value of 5.1429, an average CC of 0.2141, and an average BC of 0.3818 (see Table 2). Among them, we found the complement and coagulation cascades pathway (DC = 7, CC = 0.2381, BC = 0.5017) and PI3K-Akt signaling pathway (DC = 6, CC = 0.2518, BC = 0.5513) showed higher centrality in the target-pathway network. Meanwhile, according to copious literature, arachidonic acid metabolism, complement and coagulation cascades, PI3K-Akt signaling pathway, NF-kappa B signaling pathway, PPAR signaling pathway, cholesterol metabolism, and neuroactive ligand-receptor interaction play a significant role in CCVDs.

As a critical signaling pathway, complement and coagulation cascades involved in 7 differentially expressed genes, including F10, F3, F2, SERPINE1, F7, PLG, and PLAU. The complement and coagulation systems are two related protein cascades in plasma that serve important roles in host defense and hemostasis, respectively [55]. Recently, it has been shown that hyperactive complement is referred to the pathogenesis (including thrombosis) of diseases such as paroxysmal nocturnal hemoglobinuria, atypical haemolytic uremic syndrome, antiphospholipid syndrome, and bacteremia [56]. Upon vascular injury, tissue factor (TF) is exposed to the blood. TF exposure can initiate a series of positive feedback resulting in thrombin generation and fibrin formation, such as F3 (tissue factor) can activate F2 (thrombin) by Fxa (coagulation propagation). Ultimately, this leads to generation of fibrin, and results in the occurrence and aggravation of thrombosis. Rougui-fuzi may be used to prevent and treat CCVDs by inhibiting F2 and F3. Additionally, serine protease inhibitors (SERPINs) rapidly and irreversibly inhibit the coagulation enzymes and in concert with protein C system, ultimately terminate the coagulant response [57], which was also consistent with the rougui-fizi anti-CCVDs by inhibiting complement and coagulation cascades pathways.

Using pathway enrichment analysis, we found 5 differentially expressed genes involved in arachidonic acid metabolism, such as PTGS2, PTGS1, PLA2G2A, LTA4H, and ALOX5. The arachidonic acid (ARA) metabolic network produces a large family of inflammatory mediators, including leukotrienes (LTs) and prostaglandins (PGs), which contribute to numerous inflammatory-related diseases such as asthma and atherosclerosis. Cyclooxygenase enzymes work in one arm of the pathway to produce prostaglandins (PGs), while the actions of 5-lipoxygenase (ALOX5) and its associated protein work in the other arm of the metabolic pathway to produce leukotrienes (LTs) [58]. After conducting a literature search, the only 5-LOX inhibitor is zileuton (trade name Zyflo) in clinical trials for treating CCVDs. Inhibitors of LTA4H have also been developed [59]. We presumed that during CCVDs, the production of LTA4H, PTGS2, and PTGS1 was decreased by inhibiting ALOX5, and rougui-fuzi protected against CCVD damage by inhibiting these pathways [60].

In addition, PI3K-Akt signaling pathway referred to 6 differentially expressed genes, including IFNB1, IL4, INS, PTEN, RXRA, and TLR4, and the PI3K-Akt signaling pathway is an important signaling pathway involved in the neuroprotection against ischemic brain damage [61]. Some studies have shown that activation of the PI3K/Akt signaling pathway is related to the protection of ischemia/reperfusion damage in cardiomyocytes [61, 62]. Among them, suppressing PTEN provides protection against ischemic neuron death through both the enhancement of Akt activation and the inhibition of NR2B subunit-containing N-methyl-d-aspartate receptors [63]. PTEN inhibitors are administered prior or after experimental stroke used to acute neuroprotection following cerebral ischemia [64]. Therefore, we speculated that rougui-fuzi might be anti-CCVD by inhibiting PI3K-Akt signaling pathway.

Consequently, it was concluded that rougui-fuzi may mainly fight CCVDs through complement and coagulation cascades, arachidonic acid metabolism, and PI3K-Akt signaling pathway.

### 3.8. Protein-Protein in Interaction (PPI) Data

**Figure 8:**
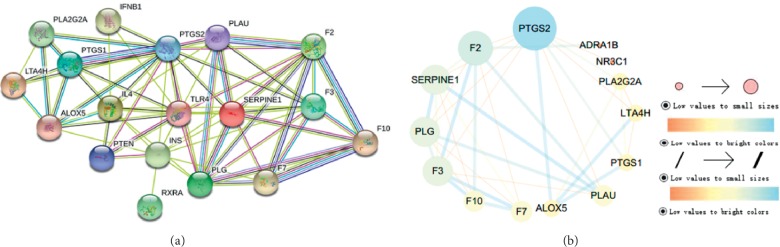
Protein-protein interaction (PPI) network by Cytoscape 3.7.1 software. The node size and color represent the size of the degree. Node size is proportional to its degree; node color is from orange to blue, and the corresponding degree gradually larger. Line thickness indicates the strength of data support. (a) PPI by string tool. (b) PPI by Cytoscape tool.

We imported 16 targets of the above three pathways into the String database, and the species was set to “*Homo sapiens*,” thereby obtaining protein interactions. Subsequently, the PPI network was further visualized and topology analyzed using Cytoscape 3.7.1 (see Figure 8). The network was constructed including 18 nodes and 67 edges. All the information of targets in PPI network was supplemented (see [Supplementary-material supplementary-material-1]). The 8 target genes with the degree greater than their average score of connectivity were selected as the hub genes for CCVDs (see Table 3), including PTGS2, IL-4, TLR4, F3, PLG, SERPINE1, and F2. According to literature research, PTGS2 rs20417CC genotype was significantly higher in patients with plaque compared with patients without plaque, and PTGS1 catalyses the conversion of arachidonic acid (AA) into prostaglandin H2 intermediates and thromboxane A2 (TxA2) [65]. Compared with healthy controls, hypertensive patients possess enhanced F2 generation, and diastolic blood pressure level of hypertensive patients is independently correlated with increased F2 generation [66]. PLG is a precursor of plasmin that degrades fibrin [67]. Plasminogen-dependent proteolysis has a beneficial effect during neurological recovery after stroke by facilitating axonal remodeling in the denervated spinal cord [68]. Based on the above, rougui-fuzi can regulate PTGS2, F2, and PLG in treatment with CCVDs.

### 3.9. Drug-Target Interaction Validation

**Figure 9:**
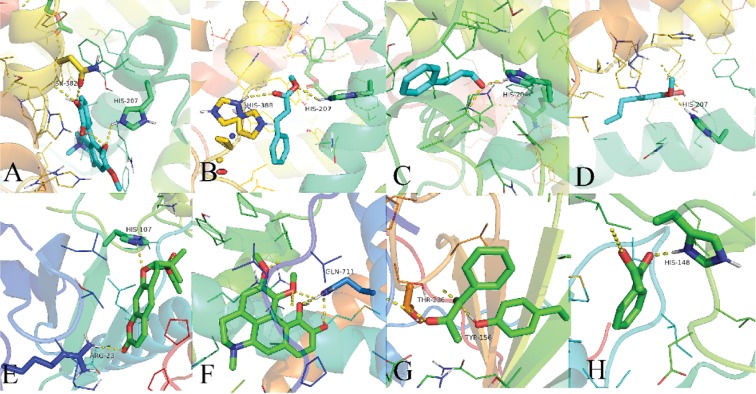
The conformations of some compounds and kernel targets: (a) PTGS2 with fuzitine, (b) PTGS2 with methylcinnamate, (c) PTGS2 with eugenol, (d) PTGS2 with cinnamaldehyde, (e) F2 with deltion, (f) AR with fuzitine, (g) F3 with pyruvophenone, and (h) F2 with pyruvophenone.

The binding energy would have many results owing to different conformations during the molecular docking. In our previous studies, the potential protein targets of TCM were successfully predicted with a good compound-protein binding affinity based on the threshold level of ≤ −5.0 kcal/mol. Thus, in the present work, target docking scores ≤ −5.0 kcal/mol as an empirical threshold were also selected as the potential targets for further analysis [43, 69]. As a result, the partial compound-target interactions with binding energy ≤ −5.0 kcal/mol were shown vividly in (Figure 9), including (a) PTGS2 with fuzitine, (b) PTGS2 with methylcinnamate, (c) PTGS2 with eugenol, (d) PTGS2 with cinnamaldehyde, (e) F2 with deltion, (f) AR with fuzitine, (g) F3 with pyruvophenone, (h) F2 with pyruvophenone. A shows that fuzitine had hydrogen bonding contact with HIS-207 and ASN-382 residues of PTGS2. B shows that methylcinnamate had hydrogen bonding contact with HIS-388 and HIS-207 residues of PTGS2. Similarly, deltion also showed its interactions with ARG-23, HIS-107 residues of the target F2 in E.

### 3.10. UPLC-Q-Exactive-MSE of Rougui-Fuzi Extracts

**Figure 10:**
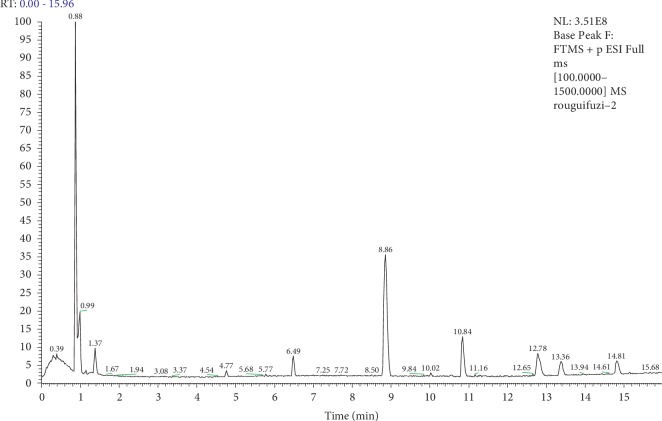
UPLC-Q-Exactive-MS^E^ total ion current chromatogram of the extract of rougui-fuzi under positive ion mode.

For explore the active chemical of the rougui-fuzi, the high resolution spectrometry and the data of mass provided sufficient information. The data obtained from an online UPLC-Q-Exactive and MS^E^ satisfied the basic needs for the analysis of complex samples by recording exact mass data for each detectable compound and its structure. The herb components were detected and finally been confirmed by MS^E^ substructure data of rougui-fuzi extracts as shown in Figure 10, including aconine, mesaconitine, benzoylhypaconine, hypaconitine, cinnamic acid, cinnamaldehyde and coumarin, and senbusine c. Among them, cinnamic acid, cinnamaldehyde, and coumarin are contained in the 21 key compounds that are obtained based on the C-T-D network for rougui-fuzi in treatment with CCVDs. The retention times, molecular formulas, molecular ion peaks, adduct ions, and cleavage fragments of the identified compounds are provided in Table 4.

### 3.11. GC-MS/MS Analysis of Rougui-Fuzi Extracts

**Figure 11:**
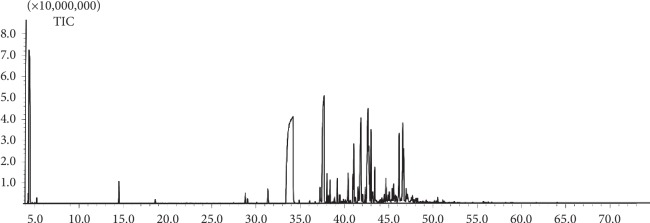
GC-MS total ion current chromatogram of the extract of rougui-fuzi.

The total ion chromatography (TIC) of essential oil of rougui-fuzi is shown in Figure 11. The analysis of GC-MS/MS data was performed using GC-MS solution software packages included with NIST14 database. 12 volatile compounds were identified based on the NIST 14 mass library by similarity searches (see Table 5), such as pyruvophenone, (L)-alpha-terpineol, cinnamaldehyde, copaene, alpha-humulene, guaiene, alpha-cubebene, 3-methoxycinnamaldehyde, *δ*-guaijene, T-cadinol, T-muurolol, and oleic acid. All these compounds belong to the 84 potential active ingredients that are obtained by TCMSP, and oleic acid, *δ*-guaijene, terpilene, (L)-alpha-terpineol, pyruvophenone, cinnamaldehyde, and 3-methoxycinnamaldehyde are contained in the 21 key compounds that are obtained based on the C-T-D network for rougui-fuzi in treatment with CCVDs. As a result, 10 of 21 key compounds have been verified by UPLC-Q-Exactive-MS^E^ and GC-MS/MS.

## 4. Conclusions

In summary, we used system pharmacology method to analyze the multicomponent, multitarget, multichannel mechanism for rougui-fuzi in treatment of CCVDs, including compound database construction, target fishing, disease mapping, network construction, and analysis. As a result, 21 key compounds, 8 key targets, and 3 key pathways were obtained for rougui-fuzi. Moreover, we performed molecular docking, which validated the reliability of the interactions of chemical compounds and targets based on systems pharmacology. Finally, we used UPLC-Q-Exactive-MS^E^ and GC-MS to analyze the potential ingredients of rougui-fuzi in the treatment of CCVDs. Collectively, herbal medicines always contain huge numbers of ingredients, diversified bioactive compounds of TCM with their related multiple targets, and multiple pathways, making it difficult to uncover the mechanisms of their interactions employing only traditional experimental approaches [70]. Systems pharmacology can offer an integrated system-level method to identify multichemicals, multitargets, and multipathways of Chinese herbs, which provided an alternative to understanding the scientific therapeutic mechanisms of Chinese herbs in treating complex diseases [21, 71].

## Figures and Tables

**Table 1:** The three centralities of 21 compounds were all higher than the average in C-T-D network.

MOL ID	Molecule name	Structure	Degree centrality (DC)	Betweenness centrality (BC)	Closeness centrality (CC)
MOL000675	Oleic acid	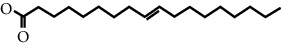	17	0.1394	0.4577
MOL002526	*δ*-Guaijene	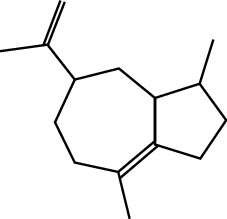	8	0.0242	0.304
MOL000911	Terpilene	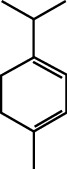	8	0.0248	0.3916
MOL000126	(−)-Nopinene	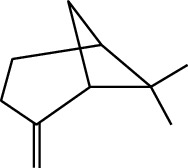	6	0.0398	0.4362
MOL000069	Palmitic acid	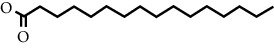	6	0.0332	0.3779
MOL000118	(L)-Alpha-terpineol	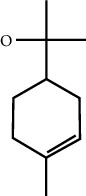	5	0.0119	0.3892
MOL000254	Eugenol	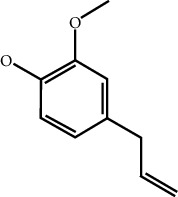	5	0.0297	0.4305
MOL003521	Isohomogenol	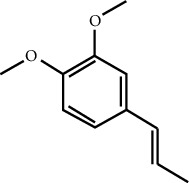	5	0.0083	0.3869
MOL000677	(1R, 4R)-4-Isopropyl-1,6-dimethyltetralin	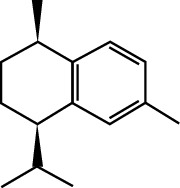	5	0.0067	0.3916
MOL002392	Deltoin	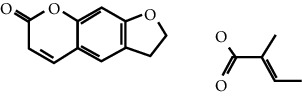	5	0.0093	0.3916
MOL002417	Fuzitine	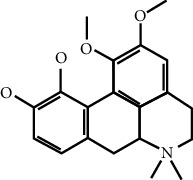	5	0.0086	0.3801
MOL000431	Coumarin	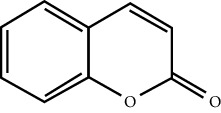	5	0.0127	0.3757
MOL003525	Pyruvophenone	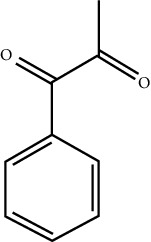	5	0.0253	0.3631
MOL000198	(R)-Linalool	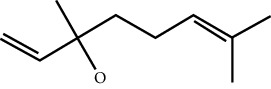	4	0.0075	0.3736
MOL000131	Linoleic acid	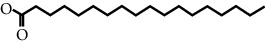	4	0.0181	0.414
MOL001393	Myristic acid	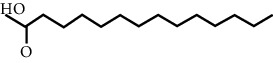	4	0.0146	0.3693
MOL000932	Alpha-farnesene	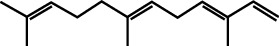	4	0.0121	0.3652
MOL000991	Cinnamaldehyde	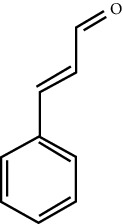	4	0.0121	0.3652
MOL002295	Cinnamic acid	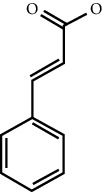	3	0.0141	0.4037
MOL000249	Methylcinnamate	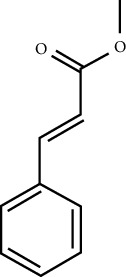	3	0.0141	0.4037
MOL003531	3-Methoxycinnamaldehyde	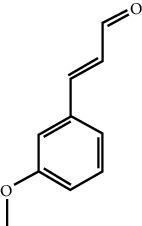	3	0.0141	0.4037

**Table 2:** The 7 pathways information of rougui-fuzi in treatment with CCVDs.

Pathways	Degree centrality (DC)	Betweenness centrality (BC)	Closeness centrality (CC)
Complement and coagulation cascades	7	0.5017	0.2381
Neuroactive ligand-receptor interaction	6	0.2193	0.1795
PI3K-Akt signaling pathway	6	0.5513	0.2518
Arachidonic acid metabolism	5	0.2185	0.2000
Cholesterol metabolism	4	0.1664	0.1515
PPAR signaling pathway	4	0.3479	0.1977
NF-kappa B signaling pathway	4	0.6673	0.2800

**Table 3:** 8 key target proteins obtained by PPI network.

Gene name	Degree centrality (DC)	Betweenness centrality (BC)	Closeness centrality (CC)
PTGS2	14	0.2411	0.8500
INS	12	0.1764	0.7727
IL4	11	0.0701	0.7391
TLR4	11	0.0734	0.7391
F3	10	0.0361	0.7083
PLG	10	0.0361	0.7083
SERPINE1	10	0.0361	0.7083
F2	9	0.0275	0.6800

**Table 4:** UPLC-Q-Exactive-MS^E^ results of the bioactive ingredients in the extract of rougui-fuzi.

Number	Compound		Area	RT (meas)	Formula	Measured m/z	Fragment ion	Delta m/z	Adducts
1	Senbusine C	+	1.2*E* + 08	6.49	C24H39NO7	454.28	436.2688, 404.2440, 409.6704	−0.05	H+
2	Aconine	+	7550000	5.73	C25H41NO9	500.285	154.9902, 182.9852, 226.9514	0.53	H+
3	Cinnamic acid	+	2260000	10.46	C9H8O2	149.06	167.1029, 362.9263, 243.9417	0.6	H+
4	Coumarin	+	8.2*E* + 07	10.55	C9H6O2	147.044	154.9902, 167.0128, 226.9513	0.36	H+
5	Mesaconitine	+	79300	12.07	C33H45NO11	632.307	124.0870, 154.9902, 167.0128	2.86	H+
6	Benzoylhypaconine	+	3.3*E* + 07	12.23	C31H43NO9	574.301	226.9514, 154.9903, 362.9263	0.15	H+
7	Hypaconitine	+	636000	12.49	C33H45NO10	616.312	226.9513, 124.0869, 167.0128	−0.26	H+
8	Cinnamaldehyde	+	2*E* + 08	13.36	C9H8O	133.065	133.0648, 154.9903, 167.0129	0.45	H+

**Table 5:** GC-MS results of the bioactive components in the extract of rougui-fuzi.

Number	RT (meas)	Compound	Formula	Molecular weight	Matching accuracy
1	18.502	Pyruvophenone	C9H80	132.15	87
2	31.402	(L)-Alpha-terpineol	C10H180	154.24	87
3	33.69	Cinnamaldehyde	C9H8O	132.15	96
4	37.633	Copaene	C15H24	204.35	95
5	40.342	Alpha-humulene	C15H24	204.35	92
6	41.463	Guaiene	C15H24	204.35	90
7	42.414	Alpha-cubebene	C15H24	204.35	90
8	42.816	3-Methoxycinnamaldehyde	C10H10O2	162.19	85
9	43.311	*δ*-Guaijene	C15H24	204.35	88
10	46.447	T-Cadinol	C15H24	204.35	94
11	46.518	T-Muurolol	C15H24	204.35	90
12	56.383	Oleic acid	C18H34O2	282.46	80

## Data Availability

The data used to support the findings of this study are included in the Supplementary Materials.

## Supplementary Materials

Supplementary Materials Table S1: 84 active ingredients of rougui-fuzi in CCVD-related diseases. Table S2: 42 potential targets of rougui-fuzi in CCVD-related diseases. Table S3: the three centralities of potential targets in the PPI network.
