# Supplementary material for: A Systems Pharmacology Approach for Identifying the Multiple Mechanisms of Action for the Rougui-Fuzi Herb Pair in the Treatment of Cardiocerebral Vascular Diseases
Source: Evid Based Complement Alternat Med. 2020 Jan 8;2020:5196302. doi: 10.1155/2020/5196302 (PMC6982690; doi:10.1155/2020/5196302)
Supplement: Supplementary Materials — Table S1: 84 active ingredients of rougui-fuzi in CCVD-related diseases. Table S2: 42 potential targets of rougui-fuzi in CCVD-related diseases. Table S3: the three centralities of potential targets in the PPI network. [file 5196302.f1.docx]

**TableS1**. 84 active ingredients of rougui-fuzi in CCVDs-releared diseases

| MOL ID | Molecule Name | Formula | Molecular weight | CAS | Source |
| --- | --- | --- | --- | --- | --- |
| MOL001123 | [Muurolene](http://lsp.nwu.edu.cn/molecule.php?qn=1123) | C10H16 | 204.39 | 99-83-2 | rougui |
| MOL000117 | [Cymol](http://lsp.nwu.edu.cn/molecule.php?qn=117) | C10H14 | 134.24 | 4939-75-7 | rougui |
| MOL000118 | [(L)-alpha-Terpineol](http://lsp.nwu.edu.cn/molecule.php?qn=118) | C10H18O | 154.28 | 10482-56-1 | rougui |
| MOL000119 | [Nerolidol](http://lsp.nwu.edu.cn/molecule.php?qn=119) | C15H26O | 222.41 | 142-50-7 | rougui |
| MOL001201 | [(1R,5R,7S)-4,7-dimethyl-7-(4-methylpent-3-enyl)bicyclo[3.1.1]hept-3-ene](http://lsp.nwu.edu.cn/molecule.php?qn=1201) | C10H16 | 204.39 | 79-92-5 | rougui |
| MOL000122 | [1,8-Cineole](http://lsp.nwu.edu.cn/molecule.php?qn=122) | C10H18O | 154.28 | 470-82-6 | rougui |
| MOL001237 | [o-Acetyltoluene](http://lsp.nwu.edu.cn/molecule.php?qn=1237) | C9H10O | 134.19 | 577-16-2 | rougui |
| MOL000126 | [(-)-Nopinene](http://lsp.nwu.edu.cn/molecule.php?qn=126) | C10H16 | 136.26 | 18172-67-3 | rougui |
| MOL000131 | Linoleic acid | C18H32O2 | 280.5 | 2197-37-7 | rougui,  fuzi |
| MOL001393 | [Myristic acid](http://lsp.nwu.edu.cn/molecule.php?qn=1393) | C14H28O2 | 228.42 | 45184-05-2 | rougui,  fuzi |
| MOL001578 | [Hypnon](http://lsp.nwu.edu.cn/molecule.php?qn=1578) | C8H8O | 120.16 | 98-86-2 | rougui |
| MOL001599 | [α-Cubebol](http://lsp.nwu.edu.cn/molecule.php?qn=1599) | C15H26O | 208.38 | 481-34-5 | rougui |
| MOL001600 | [Copaene](http://lsp.nwu.edu.cn/molecule.php?qn=1600) | C15H24 | 204.39 | 3856-25-5 | rougui |
| MOL000166 | [alpha-Bisabolol](http://lsp.nwu.edu.cn/molecule.php?qn=166) | C15H26O | 222.41 | 246-973-7 | rougui |
| MOL000170 | [Guaiene](http://lsp.nwu.edu.cn/molecule.php?qn=170) | C15H24 | 204.39 | 88-84-6 | rougui |
| MOL000172 | [Furol](http://lsp.nwu.edu.cn/molecule.php?qn=172) | C5H4O2 | 96.09 | 98-01-1 | rougui |
| MOL001739 | [Zoomaric acid](http://lsp.nwu.edu.cn/molecule.php?qn=1739) | C16H30O2 | 254.46 | 373-49-9 | rougui |
| MOL001862 | [Cadalin](http://lsp.nwu.edu.cn/molecule.php?qn=1862) | C15H18 | 198.33 | 483-78-3 | rougui |
| MOL000019 | [D-Camphene](http://lsp.nwu.edu.cn/molecule.php?qn=19) | C10H16 | 136.26 | 1422321 | rougui |
| MOL000193 | [(Z)-caryophyllene](http://lsp.nwu.edu.cn/molecule.php?qn=193) | C15H24 | 204.39 | 87-44-5 | rougui |
| MOL000198 | [(R)-linalool](http://lsp.nwu.edu.cn/molecule.php?qn=198) | C10H18O | 154.28 | 126-91-0 | rougui |
| MOL000202 | [Moslene](http://lsp.nwu.edu.cn/molecule.php?qn=202) | C10H16 | 136.26 | 99-85-4 | rougui |
| MOL000208 | [()-Aromadendrene](http://lsp.nwu.edu.cn/molecule.php?qn=208) | C15H24 | 204.39 | 489-39-4 | rougui |
| MOL002085 | [alpha-Cubebene](http://lsp.nwu.edu.cn/molecule.php?qn=2085) | C15H24 | 204.39 | 17699-14-8 | rougui |
| MOL000219 | [Acidum Benzoicum](http://lsp.nwu.edu.cn/molecule.php?qn=219) | C7H6O2 | 121.12 | 65-85-0 | rougui |
| MOL002295 | [Cinnamic acid](http://lsp.nwu.edu.cn/molecule.php?qn=2295) | C9H8O2 | 148.17 | 621-82-9 | rougui |
| MOL000234 | [L-Limonen](http://lsp.nwu.edu.cn/molecule.php?qn=234) | C10H16 | 136.26 | 5989-54-8 | rougui |
| MOL000024 | [alpha-Humulene](http://lsp.nwu.edu.cn/molecule.php?qn=24) | C15H24 | 204.39 | 6753-98-6 | rougui |
| MOL002458 | [Methacide](http://lsp.nwu.edu.cn/molecule.php?qn=2458) | C7H8 | 154.28 | 108-88-3 | rougui |
| MOL000247 | [(Z,Z)-farnesol](http://lsp.nwu.edu.cn/molecule.php?qn=247) | C15H26O | 222.41 | 4602-84-0 | rougui |
| MOL000249 | [Methylcinnamate](http://lsp.nwu.edu.cn/molecule.php?qn=249) | C10H10O2 | 162.2 | 1754-62-7 | rougui |
| MOL002502 | [Copaene](http://lsp.nwu.edu.cn/molecule.php?qn=2502) | C15H24 | 204.39 | 3856-25-5 | rougui |
| MOL002526 | δ-Guaijene, | C15H24 | 204.39 | 3691-11-0 | rougui |
| MOL000254 | [Eugenol](http://lsp.nwu.edu.cn/molecule.php?qn=254) | C10H12O2 | 164.22 | 97-53-0 | rougui |
| MOL002541 | [3-Phenylpropanal](http://lsp.nwu.edu.cn/molecule.php?qn=2541) | C9H10O | 134.19 | 104-53-0 | rougui |
| MOL000266 | [beta-Cubebene](http://lsp.nwu.edu.cn/molecule.php?qn=266) | C15H24 | 204.39 | 13744-15-5 | rougui |
| MOL002697 | [Junipene](http://lsp.nwu.edu.cn/molecule.php?qn=2697) | C15H24 | 204.39 | 475-20-7 | rougui |
| MOL002836 | [4-Methoxybenzaldehyde](http://lsp.nwu.edu.cn/molecule.php?qn=2836) | C8H8O2 | 136.16 | 68894-36-0 | rougui |
| MOL002972 | [(4S)-1-methyl-4-(6-methylhepta-1,5-dien-2-yl)cyclohexene](http://lsp.nwu.edu.cn/molecule.php?qn=2972) | C15H24 | 204.39 | 495-61-4 | rougui |
| MOL000035 | [beta-Selinene](http://lsp.nwu.edu.cn/molecule.php?qn=35) | C15H24 | 204.39 | 17066-67-0 | rougui |
| MOL003521 | [Isohomogenol](http://lsp.nwu.edu.cn/molecule.php?qn=3521) | C11H14O2 | 178.25 | 93-16-3 | rougui |
| MOL003522 | [()-Sativene](http://lsp.nwu.edu.cn/molecule.php?qn=3522) | C15H24 | 204.39 | 22469-52-9 | rougui |
| MOL003525 | [Pyruvophenone](http://lsp.nwu.edu.cn/molecule.php?qn=3525) | C9H8O2 | 148.17 | 579-07-7 | rougui |
| MOL003527 | [Tyranton](http://lsp.nwu.edu.cn/molecule.php?qn=3527) | C6H12O2 | 116.18 | 123-42-2 | rougui |
| MOL003528 | [Methylbenzofuran](http://lsp.nwu.edu.cn/molecule.php?qn=3528) | C9H8O | 132.17 | 4265-25-2 | rougui |
| MOL003529 | [m-Methylacetophenone](http://lsp.nwu.edu.cn/molecule.php?qn=3529) | C9H10O | 134.19 | 585-74-0 | rougui |
| MOL003531 | [3-MethoxycinnamalDehyde](http://lsp.nwu.edu.cn/molecule.php?qn=3531) | C10H10O2 | 162.2 | 56578-36-0 | rougui |
| MOL003534 | [Cadinene](http://lsp.nwu.edu.cn/molecule.php?qn=3534) | C15H24 | 204.39 | 523-47-7 | rougui |
| MOL003536 | [T-Cadinol](http://lsp.nwu.edu.cn/molecule.php?qn=3536) | C15H26O | 222.41 | 1474790 | rougui |
| MOL003537 | [T-Muurolol](http://lsp.nwu.edu.cn/molecule.php?qn=3537) | C15H26O | 222.41 | 19912-62-0 | rougui |
| MOL000431 | [Coumarin](http://lsp.nwu.edu.cn/molecule.php?qn=431) | C9H6O2 | 146.15 | 91-64-5 | rougui |
| MOL000475 | [Anethole](http://lsp.nwu.edu.cn/molecule.php?qn=475) | C10H12O | 148.22 | 104-46-1 | rougui |
| MOL000479 | [Farnesene](http://lsp.nwu.edu.cn/molecule.php?qn=479) | C15H24 | 204.39 | 21499-64-9 | rougui |
| MOL000489 | [(1S,4aR,8aR)-1-isopropyl-7-methyl-4-methylene-2,3,4a,5,6,8a-hexahydro-1H-naphthalene](http://lsp.nwu.edu.cn/molecule.php?qn=489) | C15H24 | 204.39 | 1460-97-5 | rougui |
| MOL002003 | [(-)-Caryophyllene oxide](http://lsp.nwu.edu.cn/molecule.php?qn=2003) | C15H24O | 220.39 | 1139-30-6 | rougui |
| MOL000057 | [1,2-Benzenedicarboxylic acid bis(2-methylpropyl) ester](http://lsp.nwu.edu.cn/molecule.php?qn=57) | C16H22O4 | 278.38 | 84-69-5 | rougui |
| MOL000608 | [()-Terpinen-4-ol](http://lsp.nwu.edu.cn/molecule.php?qn=608) | C10H18O | 154.28 | 2438-10-0 | rougui |
| MOL000612 | [(-)-alpha-cedrene](http://lsp.nwu.edu.cn/molecule.php?qn=612) | C11H12O2 | 204.39 | 103-54-8 | rougui |
| MOL000615 | [delta-Amorphene](http://lsp.nwu.edu.cn/molecule.php?qn=615) | C15H26 | 204.39 | 29350-73-0 | rougui |
| MOL000666 | [Hexanal](http://lsp.nwu.edu.cn/molecule.php?qn=666) | C6H12O | 100.18 | 66-25-1 | rougui |
| MOL000675 | [Oleic acid](http://lsp.nwu.edu.cn/molecule.php?qn=675) | C18H34O2 | 282.52 | 112-80-1 | rougui |
| MOL000677 | [(1R,4R)-4-isopropyl-1,6-dimethyltetralin](http://lsp.nwu.edu.cn/molecule.php?qn=677) | C15H22 | 202.37 | 483-77-2 | rougui |
| MOL000069 | [Palmitic acid](http://lsp.nwu.edu.cn/molecule.php?qn=69) | C16H32O2 | 256.48 | 116860-99-2 | rougui |
| MOL000698 | [(R)-(-)-alpha-Phellandrene](http://lsp.nwu.edu.cn/molecule.php?qn=698) | C10H16 | 136.26 | 4221-98-1 | rougui |
| MOL000699 | [m-Cymol](http://lsp.nwu.edu.cn/molecule.php?qn=699) | C10H14 | 134.24 | 535-77-3 | rougui |
| MOL000704 | [Styrene](http://lsp.nwu.edu.cn/molecule.php?qn=704) | C8H8 | 104.16 | 9003-53-6 | rougui |
| MOL000708 | [Phenylmethanal](http://lsp.nwu.edu.cn/molecule.php?qn=708) | C7H6O | 106.13 | 100-52-7 | rougui |
| MOL000860 | [Stearic acid](http://lsp.nwu.edu.cn/molecule.php?qn=860) | C18H36O2 | 284.54 | 8013-28-3 | rougui |
| MOL000911 | [Terpilene](http://lsp.nwu.edu.cn/molecule.php?qn=911) | C10H16 | 136.26 | 99-86-5 | rougui |
| MOL000932 | [alpha-Farnesene](http://lsp.nwu.edu.cn/molecule.php?qn=932) | C15H24 | 204.39 | 502-61-4 | rougui |
| MOL000991 | [Cinnamaldehyde](http://lsp.nwu.edu.cn/molecule.php?qn=991) | C9H8O | 132.17 | 14371-10-9 | rougui |
| MOL000012 | Arachic acid | C20H40O2 | 312.6 | 506-30-9 | fuzi |
| MOL001744 | Uracil | C4H4N2O2 | 112.09 | 66-22-8 | fuzi |
| MOL002384 | 14-Deoxy-11,12-didehydroandrographolide | C20H28O4 | 332.48 | 42895-58-9 | fuzi |
| MOL002388 | Delphin_qt | C27H31O17Cl | 662.97784 | 17670-06-3 | fuzi |
| MOL002392 | Deltoin | C19H20O5 | 328.39 | 19662-71-6 | fuzi |
| MOL002395 | Deoxyandrographolide | C20H30O4 | 334.5 | 79233-15-1 | fuzi |
| MOL002398 | Karanjin | C18H12O4 | 292.3 | 521-88-0 | fuzi |
| MOL002399 | Benzeneethanamine | C11H17NO3 | 211.29 | 54-04-6 | fuzi |
| MOL002417 | Fuzitine | C20H24NO4 | 342.45 | 142287-96-5 | fuzi |
| MOL002425 | 3-Aminophenol | C6H7NO | 109.14 | 591-27-5 | fuzi |
| MOL002427 | o-Aminophenol | C6H7NO | 109.14 | 95-55-6 | fuzi |
| MOL002428 | Azol | C6H7NO | 109.14 | 123-30-8 | fuzi |
| MOL002430 | Salsolinol | C10H13NO2 | 179.24 | 27740-96-1 | fuzi |

**TableS2**. 42 potential targets of Rougui-Fuzi in CCVDs-releared diseases

| Target Name | Gene Name | Uniprot ID | Related disease |
| --- | --- | --- | --- |
| Calcium-transporting ATPase type 2C member 1 | ATP2C1 | P98194 | Hailey-Hailey disease |
| Gamma-aminobutyric-acid receptor alpha-5 subunit | GABRA5 | P31644 | Alzheimer disease, Cognitive disorders |
| Interleukin-4 | IL-4 | P05112 | Allergy, Atopic eczema, Pulmonary tuberculosis, Idiopathic pulmonary fibrosis |
| Thrombin | F2 | P00734 | Thrombus, hypertension |
| Tumor necrosis factor | TNF | P01375 | Atopic dermatitis; Psoriatic disorder, Autoimmune diabetes,  Heart transplant rejection, |
| Mitochondrial uncoupling protein 2 | UCP2 | P55851 | Obesity |
| Plasminogen | PLG | P00747 | Bleeding, Alzheimer disease, Cerebrovascular ischaemia,  Chronic angina, Heart attack, Myocardial infarction,  Ischemic stroke, Myocardial infarction,  Coronary thrombosis, |
| Prostaglandin G/H synthase 1 | PTGS1 | P23219 | Dermatitis, Alzheimer disease, Inflammatory disease |
| Leukotriene A-4 hydrolase | LTA4H | P09960 | Myocardial infarction, Inflammatory disease |
| Prostaglandin G/H synthase 2 | PTGS2 | P35354 | Arthritis, Alzheimer disease, Inflammatory disease,  Osteoarthritis, Rheumatoid arthritis, Type 2 diabetes |
| Lysozyme C | LYZ | P61626 | Inflammatory disease, Ataxia telangiectasia |
| Glucocorticoid receptor | NR3C1 | P04150 | Ataxia telangiectasia, Rheumatoid arthritis |
| Phospholipase A2 | PLA2G2A | P14555 | Arteriosclerosis, Allergic rhinitis, Inflammatory disease |
| Superoxide dismutase [Cu-Zn] | SOD1 | P00441 | Dermatitis, Neurological disease |
| Peroxisome proliferator-activated receptor gamma | PPARG | P37231 | Stroke, Ischaemic attack, Cardicis ischaemic, Nephropathy |
| Androgen receptor | AR | P10275 | Cardiovascular disorder, Heart failure, |
| Retinoic acid receptor RXR-alpha | RXRA | P19793 | Cutaneous T-cell lymphoma |
| Alpha-1B adrenergic receptor | ADRA1B | P35368 | Hypertension, Heart arrhythmia, Psychiatric disorder, Pain |
| Phosphatidylcholine-sterol acyltransferase | LCAT | P04180 | Non-alcoholic fatty liver disease, Atherosclerosis, |
| Urokinase-type plasminogen activator | PLAU | P00749 | Cerebrovascular ischaemia, Deep venous thrombosis,  Pulmonary embolism, Myocardial infarction,  Myocardial ischemia,  Reperfusion injury, Myocardial |
| Arachidonate 5-lipoxygenase | ALOX5 | P09917 | Thrombosis, Inflammatory disease, Atherosclerosis, |
| Platelet glycoprotein 4 | CD36 | P16671 | Coagulopathy, Thrombus formation, Ischemic stroke |
| Plasminogen activator inhibitor 1 | SERPINE1 | P05121 | Thrombolysis, Cancer, Asthma |
| Myeloperoxidase | MPO | P05164 | Parkinson's disease, Inflammatory disease, Infections disease,  Chronic obstructive pulmonary disease; Multiple scierosis |
| Peroxisome proliferator-activated receptor delta | PPARD | Q03181 | Hyperlipidaemia, Lipid metabolism disorder,  Alzheimer disease |
| C-reactive protein | CRP | P02741 | Cardiovascular disease, Inflammatory disorders,  Coronary artery disease |
| Insulin | INS | P01308 | Diabetes, Metabolic syndrome x, Type 2 diabetes |
| Catalase | GCG | P021275 | Skin burns |
| Sterol O-acyltransferase 1 | SOAT1 | P35610 | Arteriosclerosis, Cardiovascular disorder,  Hyperlipidaemia, Lipid metabolism disorder,  Lipid metabolism disorder |
| Cholesteryl ester transfer protein | CETP | P11597 | Arteriosclerosis, Cardiovascular disorder,  Hyperlipidaemia, Peripheral vascular disease;  Hyperlipidemia |
| Interleukin-10 | IL-10 | Q13651 | Endometriosis, Inflammatory bowel disease;  Psoriasis, Rheumatoid arthritis,  Solid tumours, Scar tissue |
| Toll-like receptor 4 | TLR4 | O00206 | Septic shock, Prostate cancer, Hepatitis virus infection,  Autoimmune diabetes |
| Coagulation factor VII | F7 | P08709 | Factor VII deficiency, Alzheimer disease, Colorectal cancer |
| Solute carrier family 22 member 5 | SLC22A5 | O76082 | Colorectal cancer, Parkinson's, Breast cancer |
| Interferon beta | IFNB1 | P01574 | Autoimmune diabetes, Multiple scierosis, Acute lung injury, Glioblastoma multiforme |
| Dopamine D1 receptor | DRD1 | P21728 | Hypertension, Parkinson's disease, Dementia |
| Acetylcholinesterase | ACHE | P22303 | Neurodegenerative disease, Myasthenia gravis diagnosis, Epileptic seizures; Alzheimer disease |
| Nuclear factor erythroid 2-related factor 2 | NFE2L2 | Q16236 | Non-small cell lung cancer, Ocular inflammation |
| Ornithine carbamoyltransferase, mitochondrial | OTC | P00480 | Liver cirrhosis |
| Phosphatidylinositol-3,4,5-trisphosphate 3-phosphataseand dual-specificity protein phosphatase PTEN | PTEN | P60484 | Coronary micromebolization, Cancer |
| Tissue factor | F3 | P13726 | Bleeding, Pancreatic cancer, Adult respiratory distress syndrome |
| Coagulation factor Xa | F10 | POO724 | Angina pectoris, Atrial fibrillation, Venous thromboembolism, Venous thrombosis , Thromboembolic disorders, Prophylaxis of deep vein thrombosis, Deep vein |

**TableS3**. The three centralities of Potential targets in PPI network

| Number | Gene Name | Degree  Centrality  (DC) | Betweenness  Centrality  (BC) | Closeness  Centrality  (CC) |
| --- | --- | --- | --- | --- |
| 1 | PTGS2 | 58 | 0.3142 | 0.5879 |
| 2 | PTGS1 | 34 | 0.1000 | 0.4612 |
| 3 | GABRA5 | 24 | 0.0164 | 0.3591 |
| 4 | ADRA1B | 23 | 0.0384 | 0.3821 |
| 5 | RXRA | 22 | 0.0702 | 0.4147 |
| 6 | F2 | 8 | 0.0129 | 0.3520 |
| 7 | PLAU | 5 | 0.0072 | 0.3543 |
| 8 | LTA4H | 5 | 0.0018 | 0.3365 |
| 9 | TLR4 | 5 | 0.0026 | 0.3242 |
| 10 | TNF | 5 | 0.0022 | 0.3242 |
| 11 | F10 | 4 | 0.0040 | 0.3262 |
| 12 | DRD1 | 4 | 0.0017 | 0.3242 |
| 13 | NR3C1 | 4 | 0.0021 | 0.3302 |
| 14 | F3 | 3 | 0.0032 | 0.3166 |
| 15 | PLG | 3 | 0.0049 | 0.3474 |
| 16 | ALOX5 | 3 | 0.0006 | 0.3262 |
| 17 | CD36 | 3 | 0.0048 | 0.3075 |
| 18 | IFNB1 | 3 | 0.0011 | 0.3166 |
| 19 | SERPINE1 | 2 | 0.0019 | 0.3365 |
| 20 | F7 | 2 | 0.0012 | 0.3006 |
| 21 | PLA2G2A | 2 | 0.0003 | 0.3129 |
| 22 | CETP | 2 | 0.0056 | 0.3323 |
| 23 | LCAT | 2 | 0.0051 | 0.3166 |
| 24 | SOAT1 | 2 | 0.00561 | 0.3323 |
| 25 | PPARD | 2 | 0.0010 | 0.3302 |
| 26 | PPARG | 2 | 0.0010 | 0.3302 |
| 27 | IL4 | 2 | 0.0006 | 0.2956 |
| 28 | INS | 2 | 0.0023 | 0.3365 |
| 29 | PTEN | 2 | 0.0009 | 0.3185 |
